# Adverse health effects of exposure to plastic, microplastics and their additives: environmental, legal and policy implications for Israel

**DOI:** 10.1186/s13584-024-00628-6

**Published:** 2024-09-10

**Authors:** Ilana Belmaker, Evelyn D. Anca, Lisa P. Rubin, Hadas Magen-Molho, Anna Miodovnik, Noam van der Hal

**Affiliations:** 1 Faculty of Health Sciences, Ben-Gurion University of the Negev, Soroka Campus, Building M7, 8410501 Beer-Sheva, Israel; 2 Plastic Free Israel, Tel Aviv, Israel; 3 School of Public Health, University of Haifa, 199 Aba Khoushy Ave., 3103301 Mount Carmel, Haifa, Israel; 4 Hebrew University Center for Sustainability, The Hebrew University, Edmond J. Safra Campus, Givat Ram, 9190401 Jerusalem, Israel; 5 Israel Plastic Pollution Prevention Coalition (IPPPC), Tel Aviv, Israel; 6 Department of Maritime Civilizations, Charney School for Marine Science, University of Haifa, 199 Aba Khoushy Ave., 3498838 Mount Carmel, Haifa, Israel

**Keywords:** Microplastics and nanoplastics, Toxic additives, Adverse health effects, MNP in beverages, food, PCP, indoor and outdoor air, Plastic pollution, International treaties and conventions, Israeli regulations, Standards and policy

## Abstract

**Background:**

Israel is a regional "hotspot" of plastic pollution, with little discussion of potential adverse health effects from exposure to plastic. This review aims to stimulate discussion and drive policy by focusing on these adverse health effects.

**Main body:**

Plastics are synthetic polymers containing additives which can leach from food- and beverage-contact plastic into our food and beverages, and from plastic textiles onto our skin. Plastics persist in the environment for generations, fragmenting into MNPs: Micro (1 micron–5 mm)-Nano (1 nm–1 micron)-Plastic, which contaminate our atmosphere, water, and food chain. MNP can enter the human body through ingestion, inhalation and touch. MNP < 10 microns can cross epithelial barriers in the respiratory and gastrointestinal systems, and fragments < 100 nm can cross intact skin, enabling entry into body tissues. MNP have been found in multiple organs of the human body. Patients with MNP in atheromas of carotid arteries have increased risk of a combined measure of stroke, cardiovascular disease, and death. Toxic additives to plastics include bisphenols, phthalates, and PFAS, endocrine-disrupting chemicals (EDCs) which cause dysregulation of thyroid function, reproduction, and metabolism, including increased risk of obesity, diabetes, endometriosis, cancer, and decreased fertility, sperm count and quality. Fetal exposure to EDCs is associated with increased rates of miscarriages, prematurity and low birth weight. There is likely no safe level of exposure to EDCs, with increasing evidence of trans-generational and epigenetic effects. There are several existing Israeli laws to reduce plastic use and waste. Taxes on single-use plastic (SUP) were recently cancelled. There are many gaps in regulatory standards for food-, beverage- and child- safe plastic. Existing standards are poorly enforced.

**Conclusion:**

Reduction in production and use of plastic, promotion of recycling and reduction of leaching of toxic additives into our food and beverages are essential policy goals. Specific recommendations: Periodic monitoring of MNP in bottled beverages, food, indoor air; Strengthen enforcement of standards for food-, beverage-, and child-safe plastic; Renew tax on SUPs; National ban on SUP at public beaches, nature reserves and parks; Ban products manufactured with MNP; Increase research on sources and health outcomes of exposure to MNP and EDCs.

## Background

In the past year, there has been active debate in Israel over measures to reduce use of single-use plastic (SUP). Unfortunately, the debate centered primarily on the fairness of taxes on SUP while ignoring potential adverse health effects from exposure to SUP and other plastics, their disintegration products (Micro-Nano-Plastics: MNP), additives and contaminants. In this review, we focus on these health effects in order that future policy discussions and proposals will consider not only the environmental damage caused by plastics, but also their potential adverse health effects.

We present potential dangers to health from MNP, which can enter our bodies through various exposure routes including inhalation, eating, drinking and touch.

**Fig. 1:**
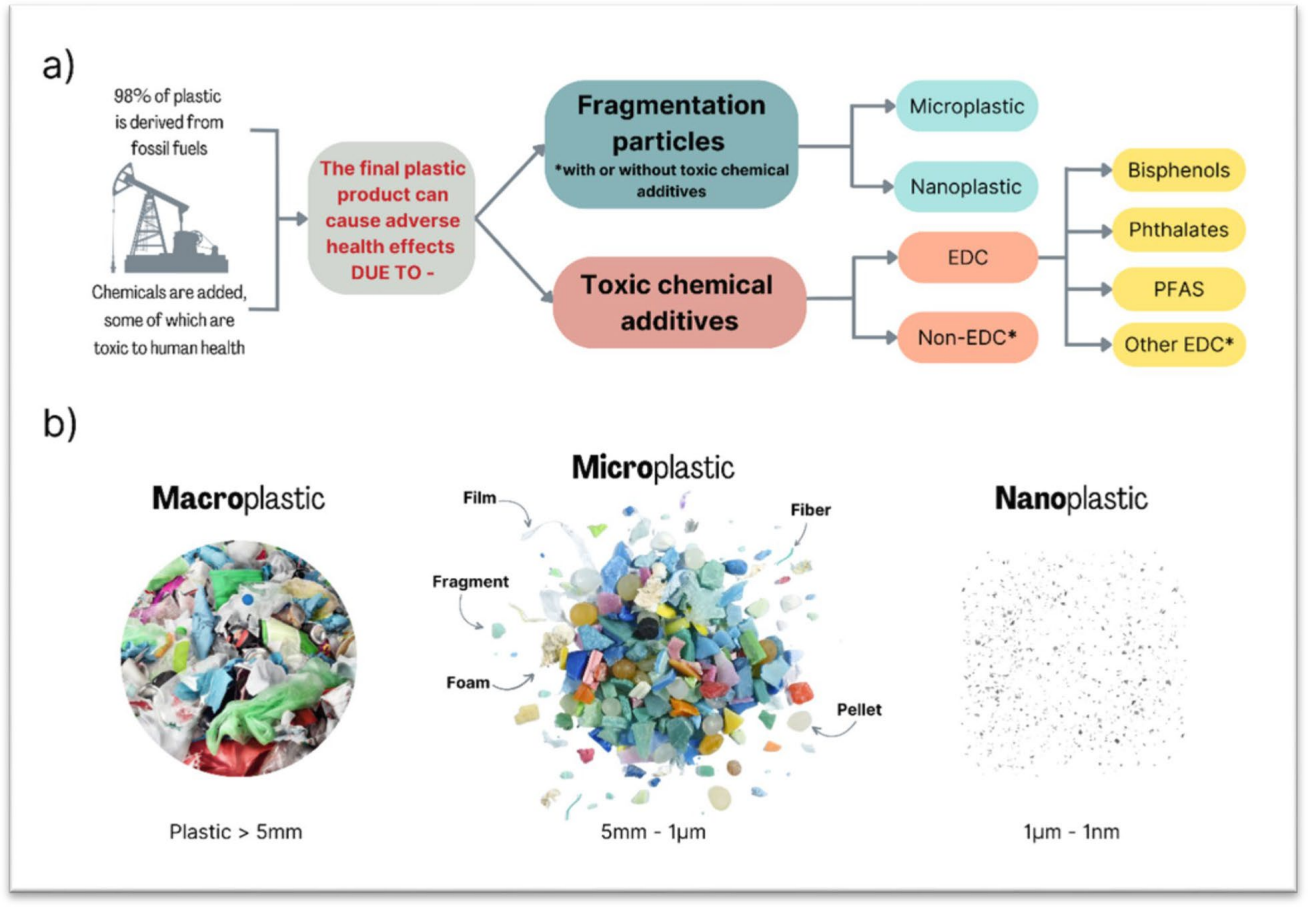
**a** Flowchart representing components of plastic of concern to human health; **b** Classification of plastic and fragmentation particles by size and shape. *EDC* Endocrine disrupting chemicals; *PFAS* Per-and Polyfluorinated Substances. Citations: **a** [1–4]; **b** [5]. *Chemical additives not covered in this article

**Fig. 2:**
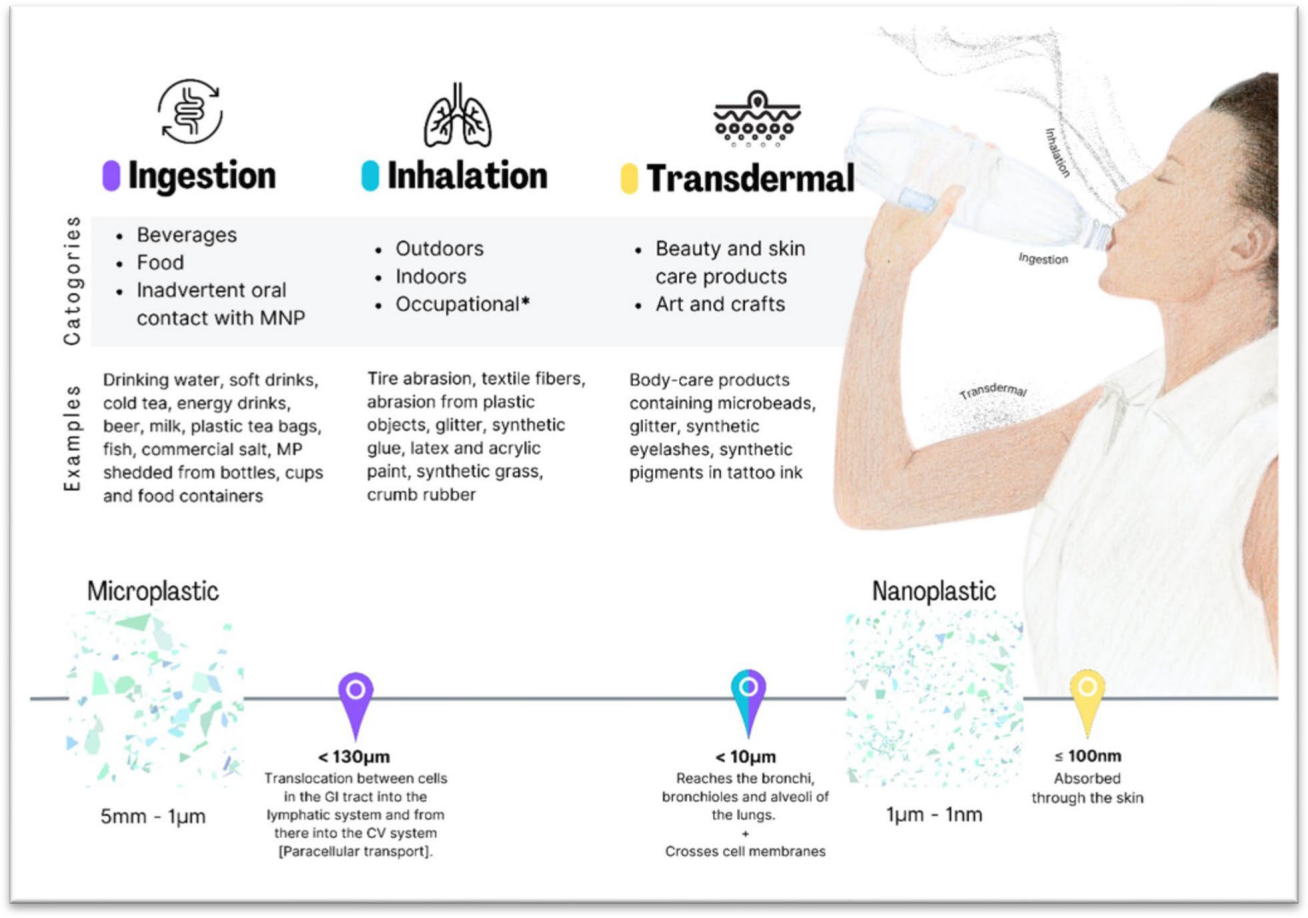
Sources of exposure to MNP and exposure line indicating the sizes that can cross epithelial barriers of the GI tract, respiratory tract, and skin. Citations: Ingestion [26–36]; Inhalation [17–20, 26, 37–43]; Transdermal [26, 40, 44, 45]; Exposure line [26, 40, 44–46]. * Not covered in this paper

We also review adverse health effects of common classes of additives to plastic and emphasize the potential for additives to plastic to leach into food and drink from plastic containers used for storing, heating, and serving food and beverages, as well as from plastic fabrics and personal care products (PCP) (See Figs. 1 and 2).

To evaluate adverse health risks of exposure to plastic, microplastic or their additives, there needs to be evidence of exposure. We present that evidence in this paper. We also summarize the growing body of research based on laboratory, epidemiological and pathological studies that point to serious adverse health effects from our widespread exposure to plastics, microplastics and their additives.

As the basis for policy recommendations, we have included sections detailing the extent of plastic and MNP pollution in Israel, as well as sections summarizing international treaties and conventions, Israeli laws, regulations, and standards, along with the activities of Israeli NGOs to decrease plastic pollution. Finally, we present policy recommendations to decrease the public's exposure to adverse health effects from plastic, MNP and their additives.

## Main text

### What is plastic?

"Plastic" is not one substance, but a class of polymers that are usually synthesized from fossil fuels. Their properties of flexibility, durability, lightweight, water repellency, and low cost have led to their use in virtually every aspect of our lives. Plastics have become one of the most ubiquitous materials in use world-wide over the past 70 years [6]. Most plastics are chemically inert and highly resistant to decomposition by the natural environment, with decomposition rates ranging from months to hundreds of years [7]. Rather than decompose, many plastic objects and waste break into smaller and smaller fragments of varying geometrical forms, called microplastic (MP) if they are 1 micron to 5 mm in diameter and nanoplastic (NP) if they are between 1 to 1000 nm (< 1 micron) [5].

MP can be directly manufactured as microbeads, and micropellets for the making of various commercial products, in addition to being formed by fragmentation of macroplastic [7].

In this paper, we use the term MNP for all microplastics less than 5 mm, unless there is a finding that relates to MP or NP only (See Fig. 1).

The final plastic product bought by the consumer typically contains additives such as stabilizers which prevent degradation of the plastic and thereby also prevent degradation of plastic waste [2].

Other additives add qualities of flexibility ("plasticizers"), flame retardation, pigmentation, anti-oxidation, and the ability to repel water and/or grease, to list the qualities of just of few of the many additives [3, 4]. Manufacturers do not label the plastics we buy with a full list of their chemical composition, including additives [8, 9].

Few countries require prior toxicological analysis of possible adverse health effects of new plastics and chemical additives that enter the global market. The European Union has regulations under its REACH program requiring the testing of new chemicals for human toxicity before their approval for marketing [9, 10]. The US Environmental Protection Agency (EPA) has authority, under the Toxic Substances Control Act (TSCA), to require such testing, if available data on a chemical are insufficient to reasonably evaluate its health and/or environmental effects [11]. However, the rate of testing does not keep pace with the rate at which new chemicals are developed and marketed. There is a growing body of literature on toxicity of certain additives to plastic. We elaborate on their adverse health effects in Sects. "Additives to plastic" and "Adverse health effects of EDC additives to plastic".

Plastic waste can form mats which float in aqueous environments. The plastic mats tend to accumulate contaminants, including persistent organic pollutants (POPs), heavy metals, algae, fungi and bacteria, including pathogenic bacteria [7]. Literature indicates that contaminants can potentially affect a variety of species, especially marine ones [12–14], yet literature with empirical data documenting the extent and health consequences of exposure to contaminants of plastic in humans is scarce [15, 16].

#### Exposure to microplastics

MNP have wide dispersion in the environment. They have been found in the atmosphere, oceans and rivers, rain and snow, dust and soil, wastewater of sewage treatment plants and in the air surrounding plastic recycling facilities [17–20]. MNP are found in in-door and out-door air, beverages, food and plastic objects we touch (See Fig. 2).

MNP smaller than 10 microns can cross epithelial barriers in the respiratory and gastrointestinal (GI) systems while only NP can cross the dermal barrier. Their ability to enter tissues of the body are size-dependent, with small particles (< 10µm) having the potential to cross epithelial barriers of the alveoli of the lungs and the GI tract and enter body tissues, including the blood and lymphatic systems [21–23] (See Fig. 2).

Larger MP are excreted from the GI tract in feces and from the respiratory tract via snot, sputum, sneezing and coughing [24, 25].

There also exists a paracellular transport mechanism in the lungs and GI tract that allow the transport of MP particles, less than 130 µm in size, across the epithelium into body tissues [26].

Data on human exposure to MNP are an essential part of risk-analysis of possible adverse health effects from exposure to MNP. We present the current state of knowledge of the extent of human exposure to MNP in the following paragraphs.

MNP are found in both out-door and indoor air, with higher concentrations in indoor air [17, 26, 37–40]. In the first study of airborne fibers in 2017, 1/3 of indoor fibers were found to be composed of plastic, while the other 2/3 were composed of natural materials [17]. Up to 60% of plastic particles in indoor air are microfibers, primarily from synthetic fabrics in clothing, textiles and padded furniture [38–40].

A recent systematic review, published in 2024, summarized all the studies since 2017 which presented data on MP in both indoor and outdoor air [47]. Indoor inhalation exposure to MP was over 3 times higher than that from outdoor inhalation exposure in all age groups. Calculated indoor inhalation exposure to MPs, using active sampling, was greatest for infants (550 MPs/kg-BW/day), followed by pre-school children, middle-school children, pregnant women, adolescents, and non-pregnant adults (152 MPs/kg-BW/day). Highest values for all age groups were found in roadside samples taken in areas of dense traffic [47]. Methodology enabling measurement of air-borne NP was not available to the authors of the studies reviewed, leading to underestimation of human exposure to inhaled MNP particles.

As shown in Fig. 2, MNP has been found in food and beverages. A thorough review in 2023 presents several factors which contribute to the contamination of food with MNP [26]: Industrial processing of food; Contact of food with plastic packaging or storage bins; Atmospheric MNP; Contact of agricultural crops with soil, fertilizer, and/or irrigation water contaminated with MNP; Contamination of the environment of marine and fresh-water fish and shellfish with water contaminated with MNP; Contact with contents of the GI tracts of animals.

Beverages are another source of exposure to MNP. In a study of 11 global brands of drinking water bottled in plastic containers [48], 93% contained MP, with an average of 325 MP particles/liter. 95% of the MP were 6.5–100 microns in size. The concentration of MP in the bottled water was double that in tap water, indicating that MP contamination can be produced by the process of bottling, or the release of MP upon opening the plastic cap on the bottled water.

Recently, a method was developed which enabled measurement of NP in water. Using this method, concentrations of MNP in water bottled in plastic containers were 2.4 × 10^5^ particles per liter, of which 90% were NP [49]. Since NP can cross the GI epithelial barrier [26] and enter body tissues, the high concentration of NP in water bottled in plastic containers may be a major source of entry of NP into body tissues.

Another source of MNP in beverages are tea bags, which when steeped in hot water, release 2.3 × 10^6^ MPs and 14.7 × 10^9^ NP per cup of tea [26].

Infant formula prepared and/or stored in plastic bottles are another area of particular concern. Release of 1.3 × 10^6^ to 16.2 × 10^6^ particles of polypropylene MP into each liter of infant formula prepared in polypropylene infant feeding bottles has been found [50]. Heating the water used to make infant formula in the polypropylene bottle, heating the water in an electric kettle with a polypropylene lining, sterilization, and repeated use of the same bottle, all increased the release of polypropylene MP into the infant formula.

MP are also found in disposable plastic cups for drinking and single-use food containers for home delivery [35].

Paper cups are often considered to be a safe alternative to plastic cups when drinking hot beverages. However, paper cups have an interior lining, usually made of polyethylene plastic, which degrades into MP when exposed to water over 85 °C. These MP then leach into the hot beverage, with a release of 2.5 × 10^3^ MP into 100 ml of hot water within 15 min, along with 102 × 10^6^ "sub-micron" particles ranging in size from 100 nm to 5 µ [51].

In an article from 2019, annual MP consumption via food was estimated to range from 39,000 to 52,000 particles depending on age and sex, with estimates increasing to 74,000 and 113,000 when inhalation is considered. Individuals who drink only water and beverages bottled in plastic were estimated to be ingesting an additional 90,000 MP annually, compared to 4000 MP for those who drink only tap water [52].

Dermal exposure to MNP can occur through use of personal care products (PCP) such as cosmetics, soap, skin conditioners, toothpaste, lip balms, exfoliants, glitter, and artificial plastic eye lashes [26], particularly those which include plastic microbeads as exfoliants. PCP can contain up to 50,000 particles of MNP per gram of product [53]. Glitter is manufactured from MP and its use in cosmetics causes direct dermal exposure to MP. The dermal barrier prevents translocation of MNP across the skin and into body tissues for particles larger than 100 nm. However, larger MNP particles can translocate across abraded, cut or inflamed skin at any age [40]. Exposure to PCP can also occur via ingestion, as a certain amount of PCP can be inadvertently swallowed.

#### Childhood as a critical period for exposure to MNP

**Fig. 3:**
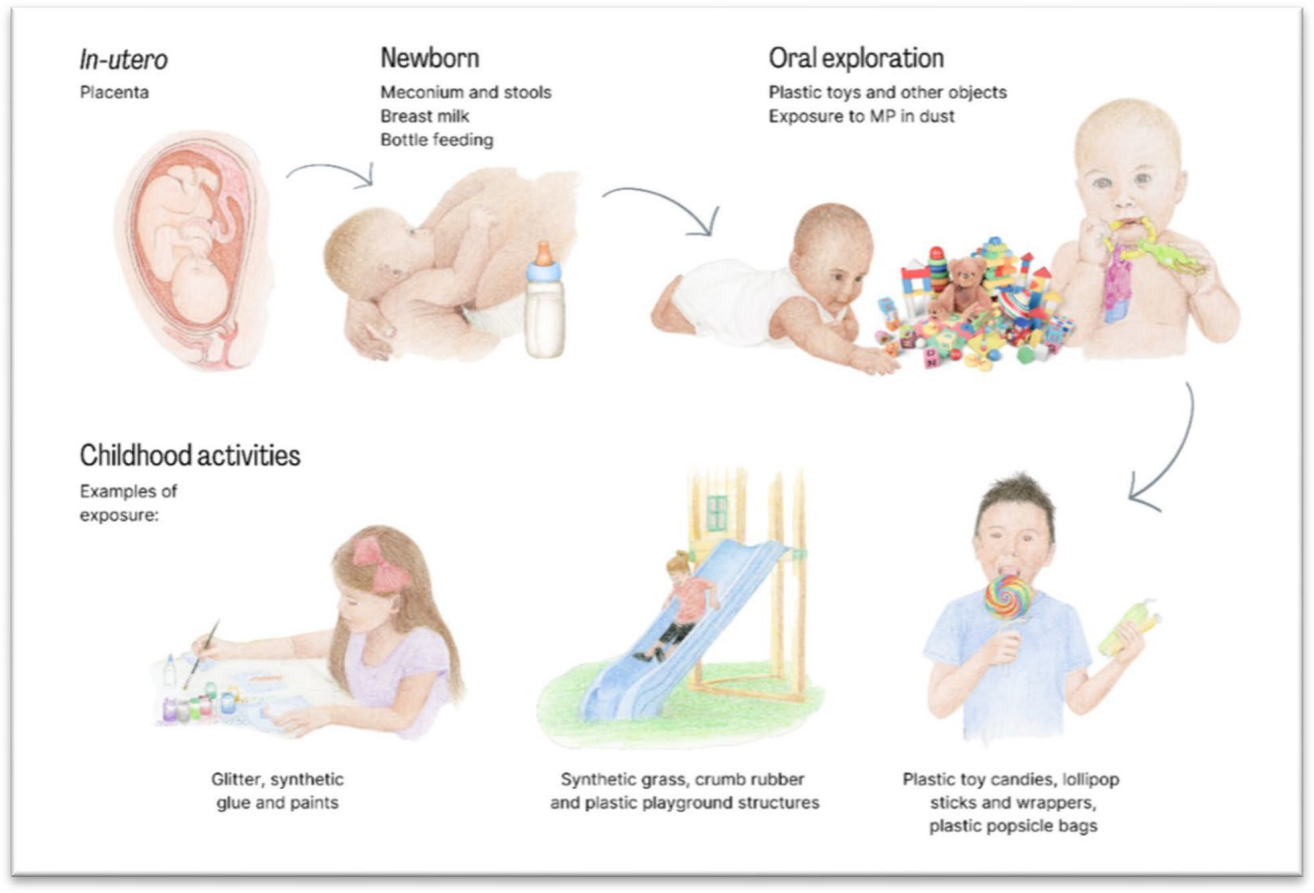
Exposure to MNP through the vulnerable stages of infancy and childhood. Citations: In utero: Placenta [54, 55]; Newborn: Meconium and infant stools [56, 57]; Breast milk: [57, 58]; Bottle feeding: [50, 57]; Oral exploration[40, 59, 60]; Childhood activities: [40–43, 61–65]

There is growing emphasis in the literature on childhood as a critical time for exposure to MNP [40] (See Fig. 3).

Exposure of fetuses in utero and newborn infants to MNP is documented by the findings of MP on the fetal side of the human placenta [55], in meconium (the feces of newborn infants who have not begun to nurse) [57], breast milk [58] and infant feces [57], in addition to polypropylene infant feeding bottles [50].

The early years of childhood are characterized by exploration of the world by crawling, picking up, tasting, and chewing plastic objects that infants encounter in their explorations. Children ingest and inhale more liquids, food, and air than adults per body weight (BW) and are thereby exposed to a higher dose level of MP/kilogram BW [40]. In a systematic review of inhalation exposures to MNP, infants were found to have the highest exposure (measured by MPs/kg-BW/day), followed by preschool children, older children, pregnant women, adolescents and non-pregnant adults [47].

The dermal barrier to environmental pollutants is not fully formed until age four, providing another route of exposure to MNP in infants and young children. Those with inflammation or abrasion of their skin are particularly vulnerable to trans-dermal absorption of MP [26, 40].

Older children can be exposed to MNP at playgrounds, as well as in their homes, daycare, and schools. Mean MP concentrations at children's outdoor playgrounds in Los Angeles were found to be 5 times greater than concentrations outside the playgrounds in the same parks [41]. More than 50% of the identified plastics were either polyethylene or polypropylene. Plastic play structures and other products used in the playgrounds, such as synthetic grass and crumb rubber, both made of MNP, can contribute to elevated MNP exposure of children playing in playgrounds [42, 43].

Infants and children are not only directly exposed to MNP, but also to toxic additives that can leach from the MNP into body tissues (See Sects. "Additives to plastic" and "Adverse health effects of EDC additives to plastic").

Since MNP do not degrade, those particles which enter the human body through ingestion, inhalation or touch, but are not excreted, can be expected to accumulate in tissues of the human body. Tissue accumulation of MP has been demonstrated in marine organisms and mammals [66]. An estimate of the lifetime accumulation of MP in children and adults has been published, taking into account both intake as well as excretion [67]**.** During their lifespan, infants and children will be expected to accumulate a larger body burden of MNP than adults, given their longer expected years of exposure and the current exponential rate of increase in production of plastic.

**Fig. 4:**
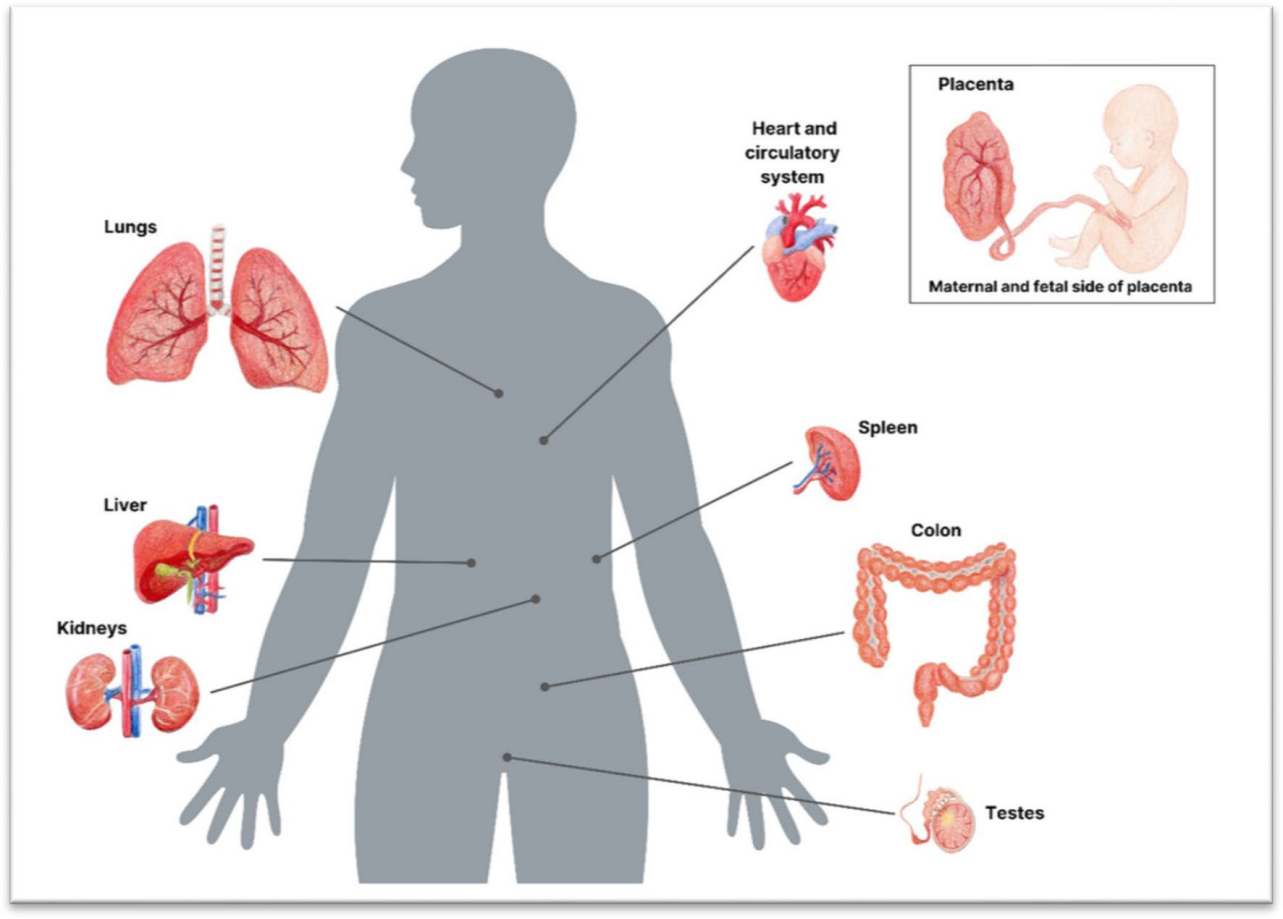
Organs of the human body in which MNP have been found. Citations: [22, 23, 54, 55, 68–71]

Given the extensive evidence of exposure to MNP from various sources, it is not surprising that MNP have been found in multiple organs of people (See Fig. 4).

MNP have not yet been found in the human brain. However, studies in mice show that polystyrene NP spheres < 50 nm in diameter can cross the blood–brain barrier in mice [72].

#### Adverse health effects of MNP

Adverse health effects of human exposure to MNP from inhalation, ingestion or touch are influenced by the size, shape and chemical composition of the plastic, additives, and chemical, bacterial and heavy metal contaminants that adsorb onto the MNP [73]. MNP can have toxic effects if additives or contaminants migrate from the microplastic into the body before excretion of the MNP occur (See Sects. "Additives to plastic" and "Adverse health effects of EDC additives to plastic").

Inflammation and oxidative stress can be caused by MNP that cross the GI, respiratory or dermal epithelial barriers and enter various organs of the body via the circulatory or lymphatic systems [74, 75]. MP, with their large surface area-to-volume ratio, cause toxicity by mechanical damage to cell membranes, DNA, and the cellular mechanisms that replicate and repair DNA. The accumulation of intracellular MP can cause chronic inflammation, with activation of the cytokine system and negative effects on the immunological systems of the body [74]. Intracellular exposure to MP can induce creation of reactive oxygen species and activation of inflammatory cells, leading to DNA damage, genotoxicity and abnormal gene expression, which is turn can contribute to carcinogenesis [76]. Additives of concern that leach out of MNP can also cause adverse health effects [3, 45, 77] (See Sects. "Additives to plastic" and "Adverse health effects of EDC additives to plastic").

Inhaled MNP contribute to the body burden of inhaled small particulate matter (PM) if they are small enough to reach bronchioles and alveoli [40]. Respiratory exposure to both PM < 10 microns (PM10) and PM < 2.5 microns (PM2.5) is associated with increased risk of asthma, chronic obstructive pulmonary disease (COPD), cardiovascular disease (CVD), and lung cancer, with causal associations between both short-term and long-term exposure and over-all mortality [78, 79]. There are studies documenting increased risk of respiratory disease and lung cancer among workers exposed occupationally to plastic dust [45, 80, 81].

The first study has just been published with direct evidence of adverse health effects from exposure to MNP [82]. A multi-disciplinary team in Italy examined carotid artery plaques ("atheromas") which were removed from 257 asymptomatic patients in order to reduce the risk of stroke. The plaques were examined for the presence of MNP. 58% of the patients had detectable levels of MNP in their plaques, with a mean concentration of polyethylene of 21.7 µ/mg of plaque. 12% of the patients also had polyvinyl chloride in their plaques, with mean concentrations of 5.2 µ/mg of plaque. All MNP visualized by electron microscopy were smaller than 1 µ (i.e. NP).

The 257 consenting patients were followed prospectively for a mean of 34 months. Those who had MNP in their plaques had significantly higher rates of a composite score of myocardial infarction, stroke or death from any cause than those who had no MNP in their plaques, with a Hazard Ratio of 4.53 (CI of 2.00–10.27) and p < 0.001.

Those with MNP in their carotid artery plaques had elevated levels of biomarkers of inflammation: interleukin-18, interleukin- 1β, interleukin-6 and tumor necrosis factor alpha (TNF-α). These findings support the inflammatory effects of intracellular MNP, as cited earlier in this Sect. [74–76].

This study is the first human study to fulfill one of the main conditions for postulating a causal relationship between exposure to MNP and an adverse health outcome: the exposure (MNP in the carotid artery plaques) occurred before the adverse health outcome. The finding of increased biomarkers of inflammation in the plaques of those who subsequently had adverse health outcomes supports the biological plausibility of the association, thus fulfilling another criterion for establishing a causal relationship. However, as the authors of the study point out, they cannot rule out that the results were confounded by other risk factors that were not included in the study design.

The recent findings of MNP in human reproductive organs (the maternal and fetal sides of placentas, testes and semen), as well as in breast milk, are a cause for concern. More research is needed to evaluate their potential threat to our reproductive system [54, 55, 57, 58, 71, 83].

### Additives to plastic

Additives to plastic of major health concern include toxic metals, such as lead, cadmium, arsenic and chromium [84], brominated flame retardants (BFR) and endocrine- disrupting chemicals (EDCs) [3]. Although there are many other additives to plastic which may have adverse health effects, we have chosen to limit this review to additives that are EDCs, since they affect not only the reproductive system, but also other aspects of our endocrine, metabolic and neuro-developmental systems. Some are carcinogenic.

We review three major additives to plastics which are EDCs: Bisphenols, Phthalates, PFAS.

#### (1) Bisphenols

Bisphenols are used in the manufacture of single-use and multiple-use polycarbonate bottles for beverages, containers for the storage of food, kitchen utensils, toys, dental sealants and even water pipes. They are used in the manufacture of epoxy resins which are used to line food and beverage cans. They can leach from food and drink containers into the food we eat and the beverages we drink [3].

The vast majority of the population of the world is exposed to bisphenol A (BPA). The half-life of BPA in the human body is short (approximately 6 h). In a study by the US Center for Disease Control (CDC), over 92% of Americans were found to have detectable levels of BPA in their urine, reflecting ubiquitous exposure to BPA. Measurable levels of BPA have been found in maternal and fetal blood and placenta and in the milk of nursing mothers [85, 86].

#### (2) Phthalates

Phthalates are plasticizers*, i.e.* they make plastic easy to mold into various shapes. They do not chemically bind to the plastic to which they are added, allowing them to migrate from a plastic item and leading to exposure via ingestion, inhalation and skin exposure. Exposure to phthalates is ubiquitous since they are found in a wide variety of products, including children’s toys, packaging for food and drinks, roofing, flooring, shower curtains and the plastic parts of cars [3].

The source of phthalates in meat and dairy products can be from exposure to phthalates in animals from which meat and dairy products are made, thereby allowing phthalates to enter the human food chain through the food itself, as well as through phthalates in the plastic packaging in which meat, fish and dairy products are marketed [87].

The phthalate DEHP is widely used in medical care products since DEHP is an integral part of the PVC which is used for the manufacture of intravenous bags, tubing, dialysis equipment, and surgical gloves.

Phthalates have a short half-life in the human body since they are rapidly metabolized and excreted in the urine. Surveys in the US have found that virtually everyone is exposed to phthalates on a daily basis. DEHP and its metabolites are present in 90–100% of samples of second-trimester amniotic fluid and are also found in cord blood of newborns, breast milk and ovarian follicular fluid [88].

#### (3) PFAS ("forever chemicals")

**PFAS**, Per-and Polyfluoroalkyl Substances, are persistent organic pollutants (POPs), chemicals that can bio-accumulate. They are known as “Forever Chemicals” due to their high resistance to chemical, physical, or biological degradation. PFAS chemicals include PFOA, PFOS, and many other fluorinated compounds. They are not found in nature, cannot be degraded by any natural organism. They cannot be metabolized in human tissues. Due to widespread environmental contamination, PFAS can be found in meat, seafood, dairy products, vegetables, fruit and water sources [89].

PFAS have grease- and water-resistant properties and are therefore found in many consumer products as additives to plastics such as polyester, nylon and vinyl fabrics, which are widely used for the manufacture of clothes, rugs, cushions, drapes and textile-covered furniture. Disintegration of these plastics allows for the creation of microfibers containing PFAS, which can be a source of human exposure to PFAS via inhalation or oral contact. PFAS can often be found in coatings of nonstick cookware, wrappers for take-away food, as well as many other consumer items. They can be found as additives to industrial equipment for the manufacture of food items and/or plastic bins in which food is stored or shipped. Since PFAS do not bind to plastic, they can migrate easily from plastic to food and beverages. PFAS has been found in some "biodegradable" bowls that are marketed as safe replacements for plastic bowls.

Due to their wide-spread use, PFAS chemicals are now ubiquitous, both in the environment and in the general population. Measurable concentrations of PFAS chemicals were found in the blood of 98% of participants, aged 12 and older, in the US CDCs National Health and Nutrition Examination Survey performed in 2003–2004 [90].

Four PFAS chemicals (PFOA, PFOS, PFNA, PFHxS) are almost universally detectable in the blood of pregnant women, neonates and children around the world, as well as in human breast milk. These chemicals can also cross the placental barrier and enter the fetus [91].

#### Adverse health effects of EDC additives to plastic

There is a vast and rapidly growing body of literature on adverse health effects of EDCs. They are chemicals called xenohormones whose structure is similar to that of naturally occurring human endocrine hormones (*e.g.* estrogen and testosterone). They can therefore cause endocrine-like effects in people exposed to them. The sources of endocrine disruptors are many and varied, including additives to plastics.

EDCs have biological effects throughout the life cycle, from the fetus, throughout infancy and childhood, adolescence, adulthood and aging [88]. However, the most vulnerable period to exposure to EDCs is the fetal period, indicating the importance of reducing maternal exposure to EDCs. Exposure to EDCs early in life is associated with childhood obesity and disorders of neurodevelopment [91]. Some of the EDCs bio-accumulate in the body, particularly in fatty tissues, while others are rapidly excreted from the body [88]. Infants can be exposed to EDCs through breast milk as well as in utero.

Of particular concern is the increasing evidence of multi-generational effects of exposure to EDCs. Exposure of a pregnant woman to EDCs can affect the health of her fetus, not only in utero but also during the growth and development of her newborn infant throughout childhood and adolescence and even into adulthood [92]. All the oocytes (“eggs”) of a female fetus are completely formed while they are still *in-utero*. Hence grandchildren can have adverse health effects from their grandmother’s exposure to EDCs via adverse effects on the oocytes of her daughter while still a fetus. There is also growing evidence that some of these adverse effects may be transmitted for more than two generations, through permanent epigenetic modifications [93].

Maternal exposure to EDCs can also affect the development of a male fetus. Maternal occupational exposure to EDCs during pregnancy has been found to be associated with poor sperm quality and concentrations in their adult sons [92]. In a recent study in mice, paternal exposure to phthalate was found to be associated with increased insulin resistance through two generations after the exposure [93].

The Endocrine Society's Authoritative Guide emphasizes that adverse health effects of exposure to EDCs can occur at very low doses and that therefore there is likely no "safe" dose of exposure to EDCs. The Guide also emphasizes that there can be a long latency between exposure to EDCs and adverse health effects [88], indicating the difficulty of identifying all the late effects of exposure. The fact that people are exposed to a varying types and concentrations of EDCs throughout life, along with varying latency periods between exposure and adverse health effects, compounds the difficulty of establishing causality between exposure and effect.

The Endocrine Society [88] and The Lancet [94] both published comprehensive reviews of literature on adverse health effects of many EDCs, including bisphenols, phthalates and PFAS, all of which are known additives to plastic. We detail these health effects below.

#### (1) Adverse health effects of bisphenols

Bisphenol A (BPA), F (BPF) and S (BPS) are those bisphenols for which there is the most evidence of adverse health effects [85]. There is growing evidence that BPA, BPF, and BPS, along with other bisphenols, can bind to estrogen, progesterone and androgen receptors and, in addition, disrupt thyroid hormone function, even at low concentrations, thereby disrupting reproduction, metabolism and neurodevelopment. Exposure to BPA is associated with altered cell division and quality of oocytes and increased incidence of polycystic ovary syndrome in women. In men, exposure to BPA and BPS is associated with decreased sperm count, concentration, quality and motility. In both sexes, steroidogenesis is adversely affected by exposure to BPA.

In adults, exposure to BPA and BPS is associated with increased risk of diabetes. In children, prenatal exposure to BPA is associated with increased levels of body fat, ADHD and behavior problems [91].

Due to all the adverse health effects associated with BPA, it has been classified by the REACH regulation of the EU as a substance of very high concern (SVHC) [88, 94].

Exposure to BPA and its analogs (BPF, BPS, BPAF, TBBPA) negatively affect reproductive health of women [95]. BPA exposure is associated with lower female fertility [96].

Analogs are increasingly used as substitutes for BPA, which has been banned in baby bottles, baby "sipping" cups and pacifiers in many countries. Since these analogs resemble BPA in their structure, there is growing concern that these substitutes for BPA may also affect the human reproductive system [95].

#### (2) Adverse health effects of phthalates

Phthalates affect estrogen and testosterone levels and function, and block thyroid action. They are therefore considered to be reproductive toxicants and classified by the EU as SVHC.

Prenatal exposure to phthalates is associated with preterm birth [97], low birth weight, childhood obesity, and impaired glucose tolerance in the mother (gestational diabetes) [98].

A recent analysis was performed of prospective studies of the associations between prenatal exposure to phthalates (as measured by gestational maternal urinary phthalates DEHP, DiDP, DnOP and DiNP) and birth outcomes (gestational age at birth, birthweight, birth length and birthweight-for-gestational age Z-scores) in 5006 mother–child dyads in 13 cohorts of diverse populations in the United States. Analysis of pooled data from the ECHO cohort study [99] found prenatal phthalate exposure to be associated with decreased gestational age and increased rates of preterm birth, with a "dose–response" relationship, i.e. higher gestational maternal phthalate concentrations were correlated with lower gestational age and higher rates of preterm births. The association was primarily driven by phthalate replacements for DEHP, which are now the main phthalates used in food packaging in the USA. The authors calculated that cost attributable to increased preterm births due to prenatal phthalate exposure in 2018 was $3.8 billion US dollars.

Male infants exposed to the phthalate DEHP during the first trimester of pregnancy have shorter ano-genital distance (AGD), while those exposed during the second trimester have smaller penile width at birth [100]. There is evidence that prenatal exposure to phthalates may be associated with increased risk of ADHD and may adversely affect psychomotor development [91].

In women, chronic exposure to phthalates is associated with decreased fertility, increased rates of miscarriages, and increased rates of complications of pregnancy, such as toxemia and preeclampsia. Exposure to the phthalate DEHP is associated with increased risk of endometriosis [88, 94].

In adult men, chronic exposure to phthalates (especially benzyl and butyl phthalates) is associated with decreased testosterone levels, decreased sperm counts and sperm quality, resulting in a decrease in male fertility. A cross-sectional study of fertile men in the US, found an association between urinary MBzP concentrations and decreased sperm motility [101].

In adults, both male and female, exposure to phthalates is associated with obesity, diabetes, and other risk factors for CVD, including elevated blood pressure, insulin resistance and elevated levels of triglycerides [88, 94].

#### (3) Adverse health effects of PFAS

In 2015 the Endocrine Society published a review of endocrine disrupting effects of EDCs [102]. They published an update of their review in 2020, adding a review of health effects from exposure to PFAS [88], while at the same time The Lancet published their systematic review of adverse health effects from exposure to EDCs [94]. Linear isomers, such as PFOS, are more toxic than branched chain polymers such as PFOA, but both can cross the placental barrier and can be found in breast milk.

Prenatal exposure to PFAS is associated with low birth weight and childhood obesity. Exposure during pregnancy is also associated with impaired glucose tolerance, insulin resistance and gestational diabetes in the mother [103–105]. Exposure of adults, both male and female, to PFAS is associated with obesity [88, 94].

In adult males, PFAS exposure is associated with lower semen quality and semen counts [106].

Some of the PFAS chemicals are carcinogenic [107]. The US National Cancer Institute has a website with current information about the association between exposure to PFAS and risk of cancer [108].

PFOA was classified as a human carcinogen in 2023 [108]. Occupational exposure and exposure through drinking water contaminated with PFOA are associated with increased incidence of kidney cancer. A study of the general population has shown that increasing serum levels of PFOA, measured before the onset of illness, are associated with increasing risk of kidney (renal) cancer. Investigators have found a possible association between PFOA and hormone receptor-negative breast carcinomas.

PFOS was classified in 2023 as a possible human carcinogen [108]. Increasing blood levels of PFOS, taken before the onset of illness, are associated with increased risk of testicular cancer. In women, elevated levels are associated with increased risk of hormone-receptor positive breast cancer.

#### Toxicology of EDCs

The testing of the toxicity of EDCs requires analysis of endocrine effects at various doses, for various periods of times, in multiple organs of exposed laboratory animals (compared with controls), as well as study of carcinogenesis. Study of the effects of maternal exposure of laboratory animals to EDCs on their offsrping includes analysis of congenital defects, growth and development (physical, neuro-behavioral, endocrinological and sexual), epigenetic effects, carcinogenesis and second-generation effects. The EDC being tested is administered to the laboratory animal at various doses, and given at various phases of pregnancy.

For a good example, see Stahl's review on the toxicology of PFAS [107]. These data from experiments in laboratory animals are then combined with knowledge from epidemiological studies in people to define a daily "No Observed Adverse Effect Level" (NOAEL). In order to provide a wide safety margin, the NOAEL level is generally divided by 100 to define tolerable daily intake (TDI) in people.

For example, The EU has classified the NOAEL for reproductive and development effects of the phthalate DEHP as 4.8 mg/kg body weight/day and the TDI (tolerable daily intake) as 0.05 mg/kg BW/day [109].

For bisphenols, in 2023 the European Food Safety Authority (EFSA) lowered its recommendations for TDI for BPA to 0.2 nanograms/kg of BW/day, a level 20,000 times lower than the TDI recommended previously in their report of 2015, based on a review of 800 new publications. The EU has also taken steps to decrease exposure to BPA via food contact materials, by setting a Specific Migration Limit (SML) of 0.05 mg of bisphenol A per kilogram of food (Regulation (EU) 2018/213), along with a ban on BPA in the manufacture of polycarbonate baby bottles (Regulation (EU) 321/2011).

In an updated 2020 recommendation regarding exposure to PFAS chemicals through food, the EFSA recommended a tolerable weekly intake (TWI) of 4.4 ng/kg body weight per week for the sum of PFOS, PFOA, PFNA and PFHxS [110].

However, the Endocrine Society, in a recent publication from 2024, together with IPEN (International Pollutants Elimination Network) states that *"*EDC exposures at even extremely low dosages can alter biological outcomes and the effects of low does cannot be predicted by the effects observed at high doses. This means there may be no safe dose for exposure to EDCs." The authors add: "EDC exposures can be harmful at any age, but they may be most harmful when people are exposed during sensitive developmental periods, such as during fetal development, infancy and childhood, adolescence and pregnancy" [111]. This strong statement throws int doubt the use of NOAEL and TDI for EDCs.

### Conditions that promote migration ("leaching") of additives from plastics into food and beverages and from there into the human body

High molecular weight phthalates (HighMWP) are used in the manufacture of PVC, which is found in food packaging and medical devices, as well as in equipment used for food processing. Food stored/prepared/served in plastic containers is therefore an important source of exposure to phthalates. HighMWP are lipophilic and therefore concentrate in high fat foods like meat, fatty fish and high-fat dairy products.

**Fig. 5:**
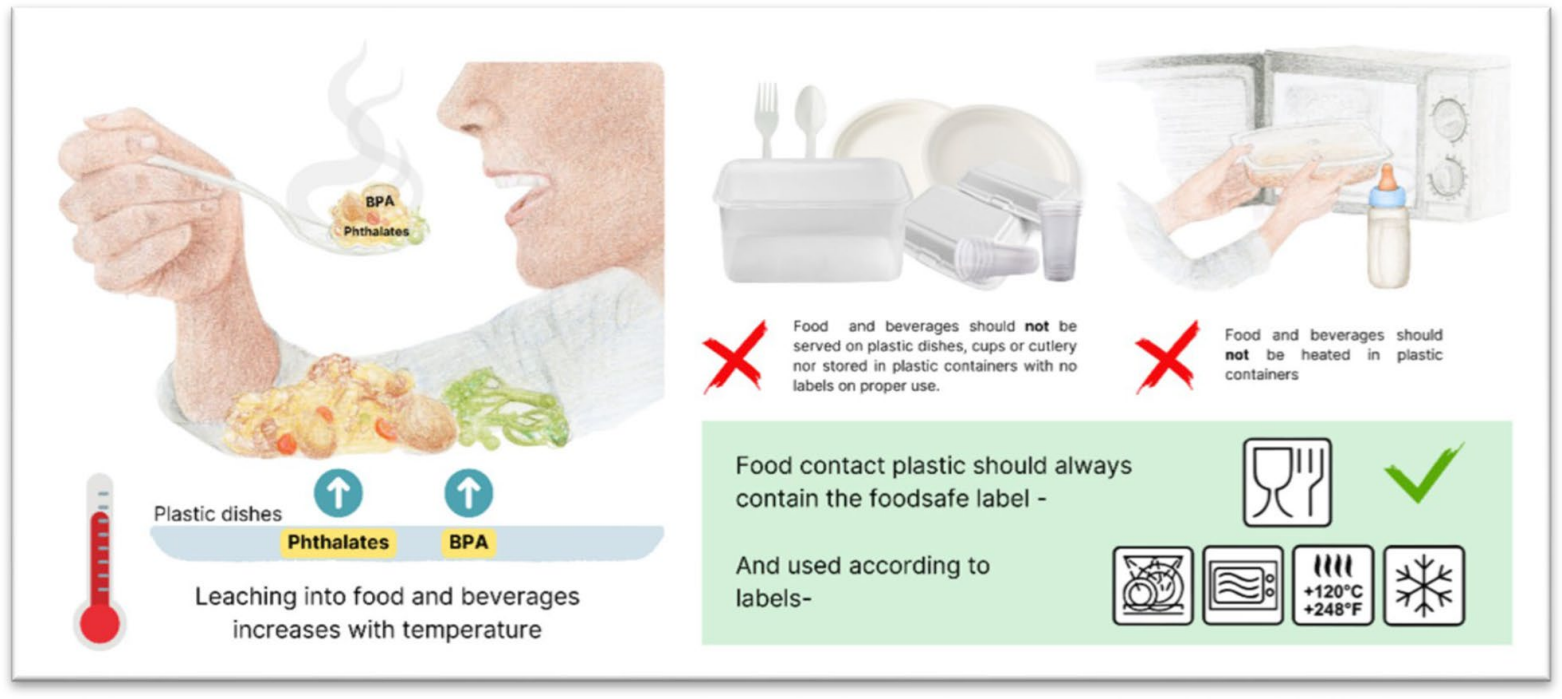
Leaching of toxic additives from plastic to food and beverages and how to reduce exposure. Citations:; Leaching [3, 84, 87, 112–114]; Do’s and Don’ts for use of food contact plastic [112]

Increased migration of phthalates and bisphenols from plastic into food and beverages is associated with heating, duration of heating, and prolonged use of plastic containers [87], as well as use of plastic items that are not certified as food-safe (See Fig. 5).

Migration of PFAS into food is promoted by high fat content of the food, low pH (i.e. acidic food), high salt concentration and emulsified food [114].

Vegetables and fruits are generally thought by the public to be free from EDCs. However, ready-to-eat vegetables, when packaged in plastic wrap, have been found to be a source of exposure to phthalates. In addition, canned fruits and vegetables can be a source of exposure to EDCs due to food contact with the lining of the cans.

Rudel studied the effect of switching from a usual diet which included packaged and canned foods to a fresh food diet, with no packaged or canned foods [113]. They recruited 20 participants from five families, who ate their usual diet, including packaged and canned food, for the first three days of the study, followed by three days in which they ate only fresh, non-packaged fruits and vegetables. In this small study, both urinary BPA and the urinary phthalate metabolite DEHP decreased significantly during the period in which participants consumed only fresh foods (reduced GM concentrations of BPA of 66% and of DEHP metabolites of 53–56%). These results support the importance of food packaging as a source of EDCs and suggest the possibility that decrease in the consumption of packaged/canned food has the potential to reduce exposure to EDCs.

Fast foods, as well as restaurant food, can be a source of exposure to EDCs. They can migrate into ingredients of food during their manufacture, storage or shipping in plastic containers, or during their preparation in the restaurant. They can also be found in food packaging used for take-away food, such as plastic food containers and wrappers.

### Israeli exposure to EDCs

A study performed in Israel in 2011–2012 found measurable concentrations of selected POPs in breast milk of Israeli mothers, including PBDE, an EDC which is added to plastic as a flame retardant [115].

In a study of urinary concentrations of BPA and phthalate metabolites in 250 Israel adults performed in 2011, detectable levels of BPA were found in 89% while detectable levels of phthalate metabolites were found in over 92%, although the concentrations found for both EDCs were below health-based threshold values at the time of the study [116]. However, the Endocrine Society has concluded that “EDC exposure and effects can occur at very low doses, below an established regulatory threshold. There is likely no ‘safe’ dose of an EDC “ [88].

In another Israeli study performed in 2015–2016, BPA and phthalates, measured in urinary samples, were obtained from 50 pregnant Israeli women. BPA was above the level of detection in 98%, and BPF and BPS were above the level of detection in 51% and 27%, respectively. Several phthalate metabolites were detected in 98%-100% of the samples [117].

Phthalates and BPA (along with trace metals and BFR) were found in a survey of soft, flexible plastic children's toys, non-PVC toys and plastic products in contact with infants such as diaper-changing mats, mattresses, feeding chairs, baby feeding aprons, baths and textiles. 65% and 10% of the items tested for two phthalates, DEHP and DINP, respectively, were found above the levels of detection (LOD), with 15% and 7%, respectively exceeding EU standards. BPA was above LOD in 22% of the items, with 17% exceeding EU migration standards for BPA. Only 1 toy, purchased on-line from India, was found to contain phthalates and BPA in levels exceeding EU standards [60].

Tordjman and colleagues studied dietary 24-h recall and urinary levels of EDCs in a pilot study of adult residents of a vegetarian community in Israel [118]. The results were compared to those found in the Israeli National Health and Nutrition Survey of 2013. Metabolites of phthalates were found in both studies, with levels of High MWP 21% lower among those living in the vegetarian community than in the national sample. Those who heated food in plastic dishes in a microwave several times a day had higher urinary concentrations of BPA (2.84 µg/g) than those who reported using a microwave no more than once per month (1.81 µg/g). Similarly, mean urinary BPA was highest among those who reported that more than 50% of their food was packed/stored in plastic (2.34 µg/g), and least among those who reported that less than 25% of their food was packed/stored in plastic (1.35 µg/g). These differences were not statistically significant, perhaps due to small sample size, but they are certainly "food for thought."

In an important review article, Berman et al. documented worrisome trends in reproductive health in Israel, with increasing rates of testicular cancer, decrease in age of menarche, increase in male infertility and decreasing sperm counts [119]. In a more recent study, Levine et al. documented world-wide decrease in sperm count at a rate that is increasing [120]. Although it is not possible to prove causal relation between exposure of populations to EDCs and changes in reproductive health of these same populations, these findings are worrisome and warrant further study.

### Plastic and microplastic pollution in Israel

**Fig. 6:**
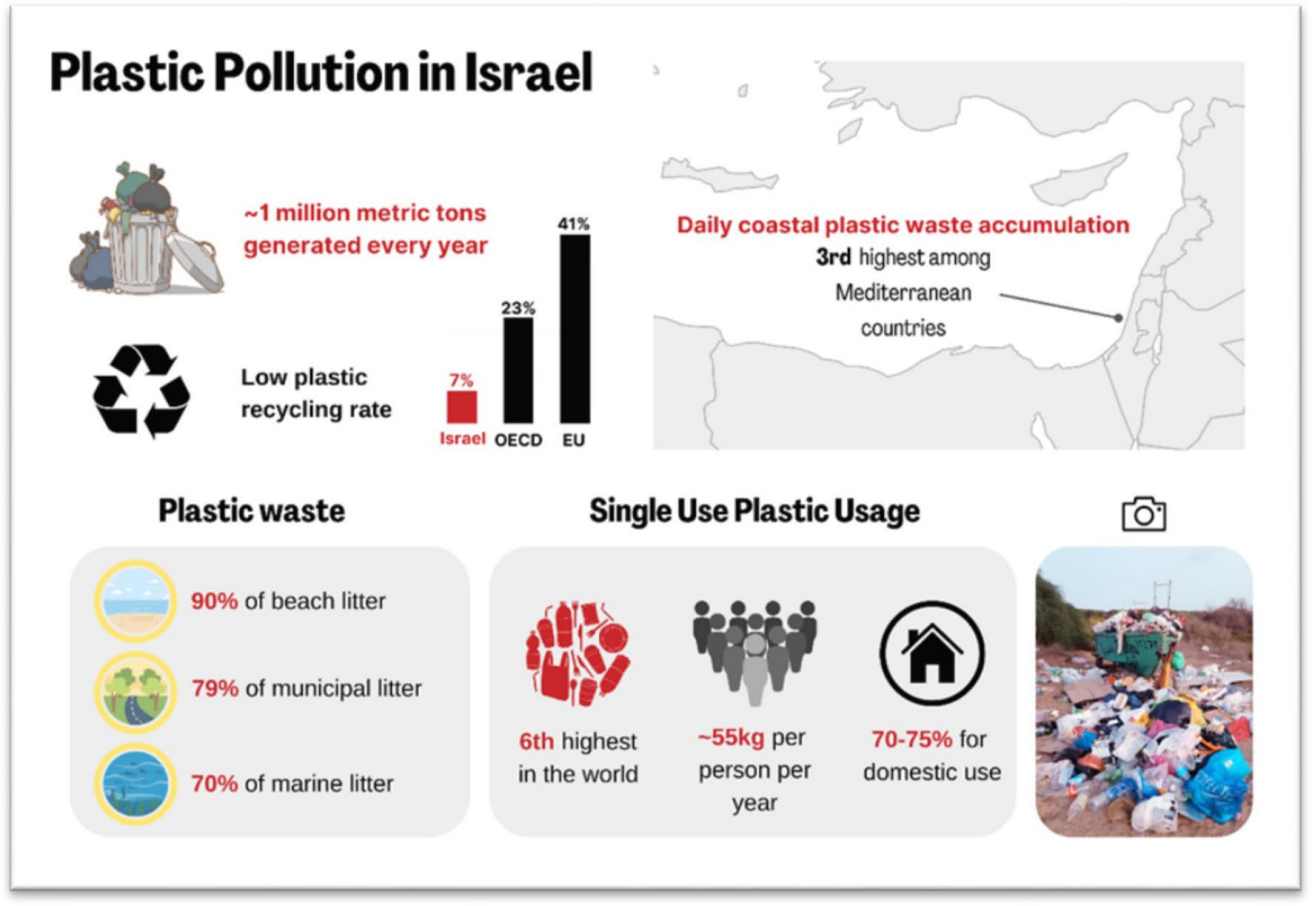
Plastic pollution in Israel. Citations: [31, 121–128]

Israel is a global and regional plastic pollution ‘hotspot’, with a continuously growing rate of single-use plastic (SUP) consumption alongside increasing pollution in public spaces and the natural environment [121–123]. Daily plastic waste accumulation on Israel’s coastline ranks 3rd highest in the Mediterranean, with 21 kg of plastic waste per kilometer of coastline [124] and is listed as a high pollution epicenter [125]. MP pollution along the Mediterranean coast of Israel is also one of the highest in the world [31, 126] (See Fig. 6).

The total amount of plastic waste created in Israel increased by nearly 16% between 2010 and 2016 and is now estimated between 900,000–1,000,000 metric tons per year [127]. Israel’s plastic recycling rate is low at 7%, compared to the OECD countries' collective average of 23% [128] and the EU, which recycled 41% of its plastic waste in 2018 [129]. A study from 2016 showed that 19,160 metric tons of plastic waste were mismanaged, namely not collected nor contained properly [130].

Israel has the sixth highest rate of SUP consumption in the world, with a 2019 average of 55 kg /year/per resident [131]**.** According to the Israel Ministry of Environmental Protection (IMEP), 70–75% of SUP are for home use, a pattern unique to Israel [121]. With over 11 billion SUP items purchased in 2020, Israeli domestic use of SUP is on the rise, has doubled in the last decade, and is now five times higher than in Europe (in kg per person)[122]. SUP is now being used by 95% of the general population, with half of the Israeli public using SUP cups on a daily basis [122]. There is rapid growth of consumption of take-away food and delivery of pre-made meals, packaged in SUP containers, to peoples’ place of work or home.

The COVID-19 pandemic has brought a worldwide increase in the use of SUP to minimize contagion risks [132]. Since the beginning of the pandemic and now, almost four years later, things are not completely “back to normal” when it comes to plastic usage, with multiple people still working from home and using delivery services [133]. The COVID-19 pandemic has increased the use of SUP in multiple sectors, worsening plastic pollution in the environment and oceans and delaying legislative efforts to reduce its consumption [134].

Plastic makes up to 41% of the domestic waste volume in Israel and 54% of it ends up in landfills [135, 136]. The high rates of SUP consumption in Israel and poor plastic waste management are often reflected in the state of public spaces and natural environments. Waste surveys from 35 municipalities show that 79% of the littered items in public spaces are plastic, mainly SUP, of which 35% are from food packaging [137]. A study conducted between 2012–2015 shows that plastic makes up 90% of the waste found on beaches in Israel, 50% of which are SUP [138]. These plastics are also found in the seabed. 70% of marine waste found in both the Mediterranean and Red Seas is plastic, mostly bags and packaging fragments [126]. After the major holidays every year, tons of plastic waste are collected from parks and along rivers and beaches of Israel [139].

The rivers in Israel are important contributors to marine pollution [140]. Littering and Illegal waste dumping are taking place in open spaces along rivers and their tributaries, which are flushed at high levels with the first intense rainfall. This contributes to contamination of water with plastic and increasing marine plastic pollution along the Mediterranean coast of Israel [141]. The Jordan river suffers particularly from pollution, contributing to plastic pollution in the Dead Sea [142, 143].

### A global overview on policies and regulations addressing plastic pollution

The connection between plastic trash discarded into the environment and human health is not intuitive. We present here 4 major pathways for plastic pollution to affect human health. (See Table 1).

**Table 1:** The connection between plastic trash discarded into the environment and human exposure to MNP, toxic additives and contaminants to plastic

A	Plastic pollution can be seen in landfills of plastic trash, as well as in piles of trash thrown in public spaces such as parks, beaches and nature preserves. The plastic trash gradually disintegrates, until it breaks down into MNP which pollute the atmosphere and contaminate the air we breathe. Other causes of the release of MNP into the atmosphere are more subtle: MNP created by abrasion of tires on roads, released into the atmosphere by ocean spray [20], fragmentation of synthetic grass [41, 42] and fragmentation of crumb rubber [43]
B	Plastic trash thrown into public spaces also enables leaching of toxic chemicals from the plastic into the ground, and from there into ground water, often used as a source of drinking water [144]. It has recently been documented that NP can migrate through soil to underground water sources, adding a new cause for concern [145]
C	Environmental plastic pollution causes fish, shellfish and marine salt to become contaminated with MNP and toxic chemicals. These enter the global food chain, and, if ingested by people, can have adverse effects on our health [146, 147]
D	The high levels of plastic pollution from SUPs indicate that much plastic is being produced in an unstainable way—Use Once and Throw Away. The cumulative amount of plastic produced since the first synthesis of plastic is estimated at 9.5 billion tons as of 2019 and predicted to increase to 34 billion tons by 2050 [148], of which most is buried in landfills, found in the depths of the ocean, or dispersed in aqueous environments, the air and land as fragmented plastic, including MNP. The rapidly growing plastic industry is highly energy-dependent and contributes significantly to global warming by its emission of greenhouse gases (GHG) [149]

#### The Plastic Treaty

The international community is tackling the increasing threat of plastic pollution to human health and the environment. In March 2022, the United Nations Environment Assembly (UNEA) adopted a resolution to launch negotiations for a new international legally binding instrument on plastic pollution: The Plastic Treaty. It will be negotiated by the International Negotiating Committee on Plastic Pollution [150]. The "Global Governance of Plastics and Associated Chemicals Report" of the UN Environment Program [151] is part of the negotiation process on the Plastic Treaty.

The goal of the Plastic Treaty is to comprehensively address plastics and their additives throughout their entire lifecycle, from production to disposal*, i.e.*, phase out or severely restrict the production and/or use of specific polymers and chemicals that are harmful to the environment and human health, are problematic in terms of impeding circularity, or that have a high risk of release into the environment. The Treaty, envisaged to be concluded by the end of 2024, would provide a comprehensive framework, building on existing international conventions.

#### International conventions

See Table 2.

**Table 2:** International Conventions that directly address plastic waste and hazardous chemicals found in plastics

The Basel Convention on the Control of Transboundary Movements of Hazardous Wastes and Their Disposal	The Basel Convention regulates and controls the transboundary movement of hazardous wastes, including plastic waste. It prohibits the export of hazardous wastes from developed countries to developing countries, unless the receiving country explicitly consents to the import. The Basel Convention also sets standards for the environmentally sound management of hazardous wastes, both domestically and internationally [152]
The Rotterdam Convention on the Prior Informed Consent Procedure for Certain Hazardous Chemicals and Pesticides in International Trade	The Rotterdam Convention regulates the international trade and safe use of certain hazardous chemicals and pesticides, including those that are used as monomers, additives, or processing aids in the production of certain plastics (*e.g.* phthalates, bisphenol A and heavy metals) It requires countries to notify each other before exporting these chemicals, and to obtain the prior informed consent (PIC) of the importing country before exporting them for first-time use. In 2019, the Parties to the Convention adopted a decision on measures to reduce the generation of plastic waste and improve its management including: promoting the use of sustainable alternatives to plastic; improving waste collection and recycling; reducing the export of plastic waste to countries that are not equipped to manage it safely [153]
The Stockholm Convention on Persistent Organic Pollutants (POPs)	The Stockholm Convention regulates chemicals that are considered persistent, bioaccumulative, and toxic (PBTs), with potentially harmful effects on human health and the environment. The Convention prohibits and restricts the use of POP additives in plastics (e.g., POPs used as flame retardants, plasticizers, or surfactants), and restricts releases of unintentional POPs (UPOPs), deriving, *inter alia,* from open burning of waste and waste incinerators. This Convention promotes the elimination or reduction of POPs through measures such as bans, restrictions, and control mechanisms [154]
The London Convention on the Prevention of Marine Pollution by Dumping of Wastes and Other Matter	The London Convention prohibits the dumping of hazardous wastes at sea. It includes stringent controls on the dumping of hazardous wastes, including plastic waste, that are likely: a) to sink to the sea bottom and remain there; b) to float or remain in suspension in the sea and interfere with navigation, fishing or other legitimate uses of the sea; c) to harm human health or marine life [155]

#### Plastics strategy and action of key OECD members

OECD member countries are taking various policy and regulatory actions to address plastic pollution [156]. See Table 3.

**Table 3:** Plastics strategy and action of key OECD members

Countries in the European Union (EU)	**Single-Use Plastics (SUP) Directive:** Bans several SUP products having available sustainable alternatives, mainly food and beverage ware, containers made of expanded polystyrene, and cotton buds. **Plastic Packaging Directive:** Sets targets for reducing plastic packaging waste and increasing recycling rates (setting mandatory recycling targets). **Packaging and Packaging Waste Directive:** Requires producers to take responsibility for the collection and recycling of their packaging waste (EPR: Extended Producer Responsibility schemes that hold producers accountable for the end-of-life management of their products). **Regulation (EU) 2023/2055 restricting synthetic polymer microparticles** on their own or intentionally added to mixtures [157–166]
Japan	**Resource Circulation Law:** Promotes the "3Rs" (Reduce, Reuse, Recycle) and EPR for plastic waste. **Promotion of Sorted Collection and Recycling of Wastes Law:** Sets recycling targets for specific materials, including plastics. Targets high recycling rates (particularly for PET bottles)[167]
Canada	**SUP Prohibition Regulations:** Bans specific SUP items (e.g., checkout bags, cutlery, straws, stir sticks). **Policy Statement on Zero Plastic Waste:** Outlines a strategy for reducing plastic waste and increasing recycling, including investments in innovation and infrastructure. Implements EPR programs [168]
USA	**Relies mainly on state and local initiatives, with limited federal regulations** (e.g. TSCA: The Toxic Substances Control Act [11] California has banned SUP bags and implemented extended producer responsibility programs for plastic packaging [169]

### Israeli laws and regulations

Israel has established legal framework and policies to manage plastic waste, microplastics, and their additives. We highlight key laws below.

The 1984 "Maintenance of Cleanliness Law" [170] for the preservation of a clean environment is fundamental legislation aimed at safeguarding the natural environment and public spaces from pollution caused by litter and unauthorized waste disposal. This law encompasses various types of waste, including plastic waste, and outlines the responsibilities of local authorities in managing designated waste disposal sites. It emphasizes that waste must only be disposed of in authorized facilities, treatment centers, or recycling facilities. Violators of this law are subject to penalties.

Section 10 of the Law establishes the "Maintenance of Cleanliness" Fund, managed by the Israel Ministry of Environmental Protection (MoEP), whose primary objective is the earmarking of financial resources for environmental protection activities in Israel, such as promotion of clean beaches, prevention of unauthorized waste disposal, and the treatment and recycling of waste. The Fund also addresses pollution caused by hazardous waste, such as asbestos and hazardous dust, which pose health risks.

#### Extended producer responsibility

One of the underlying environmental policy principles implemented in Israel is an Extended Producer Responsibility (EPR), under which a producer’s responsibility for a product is extended to the post-consumer stage of a product’s life cycle. This imposes responsibility on manufacturers and importers regarding environmental damage caused by their products, during the product’s entire life cycle. According to the principle of EPR, the negative environmental costs and the costs of dealing with waste, including collection, sorting and recycling of a discarded product, are reflected in the product price [160]. By holding manufacturers and importers responsible, Israel aims to encourage sustainable practices and minimize the environmental impact of products and their waste.

#### Key laws addressing plastic waste

In Israel, there are several EPR legislative measures, including laws specifically targeting reduction in plastic pollution and promoting sustainable practices. Below are four key laws related to plastic use and waste in Israel, which are currently in force (See Table 4).

**Table 4:** Current Israeli EPR Laws addressing plastic pollution

**Deposit Law on Beverage Containers (1999):** This Law (also known as ‘The Bottle Bill’) requires a refundable deposit to be paid on beverage containers sold in Israel, thereby promoting reuse and recycling of beverage containers, and reduction of litter in public spaces and of waste in landfills. It regulates the recycling of beverage cans and bottles made of glass, metal, and plastic with volumes ranging from 0.1 to 5.0 L. Consumers pay a deposit fee upon purchase, which is refunded upon returning empty containers. The funds collected through the law go to the MoEP Cleanliness Fund and are used to promote beverage container recycling and enforcement of environmental legislation [171]
**The "Single-Use Plastic Bags Reduction Law" (2016):** This law aims to reduce the use of SUP bags, thereby reducing the amount of waste generated and its negative environmental impact. The law requires supermarkets (at the check-out counter) to charge a fee for SUP bags. The law promotes alternatives to SUP and encourages recycling [172]
**Packaging Law (2011)** The Packaging Law places responsibility for the collection and recycling of a product's packaging waste directly upon that product's manufacturers and importers. The Law’s main goal is reduction of the negative environmental impact of consumer product packaging and prevent its landfilling, by encouraging recycling of plastic packaging of food, personal care and household cleaning products, as well as nylon bags, milk and juice cartons and empty tins of canned food. Orange plastic trash collection bins are placed in each municipal neighborhood by the municipality, which also pays for transport of the bins to a central processing center. It focuses on minimizing packaging waste generation, preventing its accumulation, and promoting packaging reuse [173]
**Tires Disposal and Recycling Law (2007):** This Law regulates the disposal and recycling of tires to protect the environment and prevent harm caused by discard of tires in unregulated sites, uncontrolled burning and landfill accumulation. This law places the responsibility for tire recycling on tire producers and importers. Tire producers and importers in Israel are responsible for collecting used tires and either recycling them or finding other ways to reuse them [174]

#### Israeli Standard 5113: ensuring safety of plastic materials in contact with food

Israeli Standard 5113 is an official regulation in Israel that is based on EU regulations and the U.S. Food and Drug Administration (FDA) for certifying the safety of food- and beverage- contact plastic [112]. In general, there is compliance with regulatory standards in Israel for children's toys and other child items with mandatory standards, but regulatory gaps leave many products untested [175].

This standard requires testing of products based on their intended use, such as microwave heating, contact with hot or cold food, or prolonged storage. The tests are designed to assess the general migration of substances from the plastic material, as well as the specific migration of hazardous substances that may pose risks to human health. This includes heavy metals like lead, copper, iron, nickel, aluminum, as well as potentially harmful organic compounds and carcinogens.

Additional important regulations: Feeding bottles for infants—Standard 5817; Pacifiers for children—Standard 1157; Feeding accessories for children—Standard 14,372; Children's jewelry—Standard 6558.

### Citizen activities in Israel to reduce plastic pollution and exposure to additives of plastic

Non-Governmental Organizations (NGOs) play a significant role in reducing plastic pollution and exposure to plastic additives in Israel. They operate individually on many levels: education; advocacy; research; policy. Many environmental NGOs have joined the Israeli Plastic Pollution Prevention Coalition (IPPPC), which facilitates cooperation and consensus building on important initiatives.

Together and individually, NGOs educate the public about the negative impacts of plastic pollution, promote alternative, environmentally friendly products, organize beach cleanups and campaigns to remove plastic waste from the environment. They also prepare position papers, press briefings, and testify before Knesset committees.

Public participation and consumer education and engagement are an essential part of developing policies that are acceptable and sustainable [176]. The cancellation of the tax of SUP is an example of the consequences of lack of broad citizen participation in the decision-making process. Even though the tax was successful in decreasing by 18% beach litter of SUP items included in the law [177], the Finance Minister of the new Israeli government, formed at the end of 2022, immediately cancelled the tax, since his voting constituents felt that it targeted them unfairly [178]. The Finance Minister subsequently has asked for recommendations for a more comprehensive plan to decrease use of and pollution by SUP. It is important that new recommendations will be drawn up with public participation from a wide range of stakeholders.

### Health policy implications of this review

We could find no literature on preventive measures to mitigate adverse health effects of MNP or toxic additives after exposure to them. In the absence of post-exposure measures, effective policy interventions need to emphasis prevention of exposure to MNP and toxic additives to plastic.

Proposed interventions, their implementation and management, require a multi-faceted approach involving government, industry, NGOs, academia, and the public at the local, regional and national levels. These health-driven policy recommendations are presented in two tables (Table 5a and b).

Our policy recommendations to reduce adverse health effects from plastic pollution are tailored to Israel's specific situation, including low public awareness of the adverse health consequences of exposure to plastic and plastic pollution and high use of SUP.

**Table 5:** (a) Policies to reduce current exposure to MNP and toxic additives to plastic

1	Expand testing and enforcement of existing Israeli standards for certification of food-, beverage- and child-contact plastic and enlarge the list of toxic chemicals tested as new data on the toxicity of various chemicals are published in scientific literature
2	Perform periodic national monitoring of EDCs and other toxic additives to plastic in a representative national sample of the Israeli population to follow trends in exposure over time, identify those populations most at risk and to tailor intervention programs appropriately
3	Identify sources of exposure to MNPs by ingestion through periodic surveys of the concentration of MNPs in beverages (tap water, bottled water, bottled beverages, milk and fruit juices), and food (agricultural products sold at both wholesale and retail levels; processed food products; food manufacturing facilities)
4	Identify sources of exposure to MNPs by inhalation through funding of periodic surveys of the concentration of MNPs in indoor air (households, classrooms, health care facilities, workplaces and indoor sports facilities) and out-door air (children's playgrounds, amusement parks, sports stadiums, sewage treatment facilities, factories for plastic recycling)
5	Promote non-plastic alternatives to synthetic grass and crumb rubber in playgrounds, parks and sports facilities to reduce exposure to MNPs by inhalation and dermal contact
6	Reduce dermal exposure to MNPs through bans on import, manufacture and sale of cosmetics, personal care products, cleaning products and glitter manufactured with MNPs
7	Require labelling of all food-, beverage-, and child-contact plastic, with name of manufacturer and compliance with Israeli standards for food-safe, beverage-safe and child-safe plastic. For food and beverage containers, labelling should include the safe temperature to which they can be heated without danger that toxic chemicals may leach into the food/beverage
8	An official website of an authoritative governmental body should be established, providing information about safe use of food-, beverage-, and child-contact plastics, with explanations about the labelling on plastic products, and a clear recommendation against heating or serving hot food and beverages in any plastic container unless it is certified as food-safe and not used at a temperature above that recommended by the manufacturer The public should be advised to avoid buying cosmetics, body care products, cleaning products or glitter containing MNP or microplastic beads (as long as there is no ban on their sale) The website should be regularly updated and have a call center or chatbot that will allow the public to ask questions
9	Public awareness campaigns should be launched, to educate the public and encourage behavioral changes to reduce exposures National and local health promotion campaigns are needed to promote safe use of plastic and encourage use of alternatives to plastic, such as glass, wood, metal, ceramics, fabrics from natural materials
10	Special health promotion programs should be implemented for those populations most likely to have exposure to MNPs or toxic additives to plastic which can affect their health or that of their children: pregnant mothers, infants and children, as well as both members of a couple who are trying to conceive. Such a program can be integrated into the preventive health care services of Israel

(b) Policies to reduce future exposure to MNP and toxic additives by reduction in plastic pollution	
1	Re-enact the tax on SUP to discourage consumption. The tax should be placed on all plastic designed to be used once and then discarded, including SUP used to package and transport take-away food, home deliveries of food, and serving of food and beverages
2	Expand the current, effective, "Single-Use Plastic Bags Reduction Law" to other retail stores
3	Pass a nationwide prohibition on the use of SUP on public beaches, nature reserves and parks
4	Transition towards a model that prioritizes reduction in plastic production, importation and sale by promotion of alternatives to plastic, along with promotion of a circular economy for plastic [179]
5	Production of less plastic and a shift towards use of renewable energy sources for plastic manufacture and recycling to reduce their contribution to global warming [180]
6	Support continuing research on environmental sources of plastic pollution in Israel, and methods for its reduction
7	Recycling plants themselves should not be a source of MNP pollution, neither air pollution nor pollution of wastewater. An official standard should be established on MNP emissions for factories manufacturing or recycling plastic, as well as wastewater treatment plants, [181]
8	Expand implementation of Extended Producer Responsibility (EPR) of producers for the entire lifecycle of plastic products, incentivizing sustainable design and recycling

## Conclusions

Although research on potential adverse health risks from exposure to plastics, MNP and their additives is at an early stage, these health risks are progressively gaining recognition. The topic covers a wide variety of exposure risks that vary by types of plastic and their additives, leaching of toxic additives out of plastics into beverages, food, and textiles, along with the non-degradable nature of plastic, causing its fragmentation into Microplastic (1 micron-5 mm) and Nanoplastic (1 nm-1 micron), collectively known as MNP. These MNP spread throughout our environment, contaminating the atmosphere, water, and land of the earth, allowing MNP to work its way up through the food chain, disrupting global ecology. Humans can be exposed to MNP via inhalation, ingestion and touch, while fetuses can be exposed via maternal exposure and infants can be exposed through breast milk.

However, it is difficult to establish causality, rather than associations, between exposure and adverse health effects [182]. A groundbreaking paper has just been published which strengthens evidence for a causal association between exposure to MNP and adverse health effects by documenting exposure to MNP (in atheromas of the carotid arteries) prior to the occurrence of an adverse health event (increased risk of a combined measure of stroke, CVD and death from any causes over a mean follow-up period of 34 months [82]. The biological plausibility of such an association was strengthened by the finding of increased levels of biomarkers of inflammation in those with MNP in their carotid arteries, since inflammation can be caused by intra-cellular MNP [74–76]. The effects of MNP on cellular toxicity, including inflammation, are supported by studies in mammals [77]. Further studies, such as this groundbreaking research [82], are needed to strengthen evidence for a causal association between exposure to MNP and adverse health outcomes.

The growing number of studies documenting the presence of MNP in various organs of the human body are a cause for concern, since they are indestructible by any biological process. Therefore, once present in human tissue, they are likely to persist. The recent findings of MNP on the fetal side of the human placenta, meconium of new-born infants, breast milk, the circulatory and digestive systems, lungs, liver, spleen, kidneys, testes, and sperm are particularly worrisome.

Over their lifespan, infants and children will accumulate a much larger body burden of MNP than adults who were born decades ago, given the current exponential rate of increase in production of plastic and their longer potential years of exposure. However, exposure does not mean that the MNP will pass the epithelial barriers of the lungs, GI systems and skin and adversely affect health. Much more research needs to be done on this topic [183]. As stated in the paper by Ramsperger et al. in 2023 [26]: "Even if only small fractions of NMP can overcome epithelial barriers, the long-term effects of persistent particles and associated chemicals should not be under-estimated."

The urgency of our problem of exposure to MNP is emphasized by the decision of the European Commission, in September 2023, to restrict intentionally-manufactured MP less than 5 mm in size, including immediate bans on glitter and microbeads, and to restrict manufacture of granular infill materials manufactured from MP that are found in both artificial grass and "crumb rubber" surfaces [161].

Plastics include many toxic additives, including EDCs, that we chose to review in this article. The toxic effects of exposure to EDCs have been widely researched, in epidemiological studies and animal models. Those studies in which exposure is measured before the adverse outcome (such as those studying the association between levels of EDCs in pregnant mothers and various measures of health, growth and development of her infant) strengthen the evidence of a causal relationship between exposure to EDCs and adverse health effects [95], [96], [97], [98], [100], [102], [103].

There is growing concern about potential adverse reproductive health effects of EDCs at the population level, with decreasing sperm counts, increasing rates of testicular cancer and decreasing age of menarche in the majority of industrialized countries around the world [119], [120].

This article reviews selected EDCs additives to plastics (phthalates, bisphenols and PFAS), which can cause adverse health effects. A recent statement by the Endocrine Society states that a paradigm change is needed regarding exposure to EDCs, since even very low exposure doses can have adverse health effects [111]. There is likely no “safe level” of exposure.

The current level of plastic production is non-sustainable and incompatible with public health. In 2021 alone, 390 million metric tons of plastic were produced worldwide [148]. The plastic industry was responsible for 3.4% of global carbon emissions in 2019 and expected to account for over 10% of the remaining carbon budgets by 2050 [184, 185]. Landfills, in which plastic disintegrates over decades and hundreds of years, emit the GHG methane, more potent than CO_2_ in its warming potential [186]. This significant GHG addition to global warming by the plastic industry is an additional threat to the health of people, both currently and in the future.

Our integrative review is geared towards promoting awareness of the Israeli public and policy makers to the growing threat of adverse effects to our health due to exposure to plastics, MNP, and their additives. It is difficult, however, to accurately assess the exact risk to human health, since all populations are universally exposed to both MNP and EDCs, measurements of exposure are difficult, and health consequences of exposure may appear only after years. EDCs can exert effects at very low doses compared to traditional chemicals, raising questions about the existence of a safe threshold of exposure. Moreover, exposure during critical developmental windows (fetal development, early infancy) may have long lasting consequences that traditional adult-based studies may miss.

In their seminal paper entitled "Why endocrine disrupting chemicals (EDCs) challenge traditional risk assessment and how to respond", the authors present innovative ideas about non-traditional ways of estimating risk assessment for EDCs [187]. However, implementation of their innovative ideas requires the development of methodology that does not yet exist.

Despite the inability at this time to perform accurate risk assessment of exposure to MNPs and EDCS, there is a growing body of literature on evidence of harmful health effects from exposure. We summarize the existing evidence for harmful effects to human health in this paper.

Our paper develops the case for applying the Precautionary Principle to slow and eventually stop the rapid increase in our exposure to plastic, MNP and their additives. The Precautionary Principle is a pro-active policy-making framework that prioritizes early intervention when there are threats of serious or irreversible damage to health or the environment, even when there is lack of full scientific certainty about potential harm. Delaying a policy decision until there is absolute scientific certainty cannot be used as a reason for postponing intervention [188, 189].

In Sect. "Health policy implications of this review" and Table 5a and b, we detail proposed actions which require a multi-faceted approach involving government, industry, NGOs, academia, and the public at the local, regional and national levels, with implementation through intertwined multiple, bottom-up and top-down approaches.

Israel has few standards and little enforcement of the quality of plastic that is manufactured, imported, or recycled and few labelling requirements. This allows unregulated plastics to enter the market, whether they are safe or not. The initiative of the EU to regulate plastics, MNP, and additives, covered in Sect. "A global overview on policies and regulations addressing plastic pollution", provides a template for future laws and regulations in Israel. The recent UN initiative to develop a legally binding international Plastic Treaty to prevent plastic pollution and exposure to toxic additives (reviewed in Sect. "A global overview on policies and regulations addressing plastic pollution" provides a note of optimism.

On the global level, the COVID-19 pandemic has increased the use of SUP in multiple sectors, worsening plastic pollution in the environment and oceans and delaying efforts to reduce its consumption [134]. A particular challenge to the health care industry is to find safe replacements for much of the plastic that is used to diagnose and treat patients [190].

Attacking the problems of plastic pollution and exposure to adverse health effects of exposure to plastics, MNP and toxic additives will require a major change in our culture and a mindset switch that emphasizes and accepts the urgency to act now to protect the health of current and future generations. We hope this integrative review can be a driving force to stimulate public debate and action.

## Data Availability

*Data from Individual Person*: Not applicable. All articles and materials used in preparing this paper can be found on the internet, as noted in the references. *Datasets*: Not Applicable.

## Abbreviations

ADHD: Attention-deficit hyperactivity disorder
BFR: Brominated flame retardants
BPA: Bisphenol A
BPAF: Bisphenol AF
BPF: Bisphenol F
BPS: Bisphenol S
BW: Birth weight or body weight
CI: Confidence interval
CO_2_: Carbon dioxide
COPD: Chronic obstructive pulmonary disease
CV: Cardiovascular
CVD: Cardiovascular disease
DEHP: Di-2-ethylhexyl phthalate
DiDP: Diisodecyl phthalate
DiNP: Diisononyl phthalate
DnOP: Di-n-octyl phthalate
ECHA: European Chemicals Agency
EDC: Endocrine disrupting chemical
EFSA: European Food Safety Authority
EPA: Environmental Protection Agency
EPR: Extended producer responsibility
EU: European Union
FDA: Food and Drug Administration of the USA
GHG: Greenhouse gases
GI: Gastrointestinal tract
HDPE: High density polyethylene
HighMWP: High molecular weight phthalates
IMEP: Israel Ministry of Environmental Protection
LOD: Level of detection
MBzP: Monobenzyl phthalate
MoEP: Ministry of Environmental Protection
NGO: Non-Governmental Organization
MP: Microplastic [1 micron (1 µ) to 5 mm in size]
MNP: All micro- and nano-plastics
NP: Nanoplastic [1 nm to 1000 nm; 1000 nm = 1 micron (1 µ)]
OECD: Organization for Economic Co-operation and Development
PBDE: Polybrominated diphenyl ethers
PCP: Personal care products
PFAS: Per-and polyfluoroalkyl substances
PFHxS: Perfluorohexane sulfonate
PFOA: Perfluorooctanoic acid
PFOS: Perfluorooctane sulfonate
PFNA: Perfluorononanoic acid
PM: Particulate matter
PM10: PM < 10 µ in diameter
PM2.5: PM < 2.5 µ in diameter
POP: Persistent organic pollutant
PVC: Polyvinyl chloride
REACH: Registration, Evaluation, Authorization and Restriction of Chemicals
SIoI: Standards Institution of Israel
SMM: Sustainable Materials Management
SUP: Single use plastic
SVHC: Substance of very high concern
TBBPA: Tetrabromobisphenol A
UN: United Nations
USA: United States of America
